# Methodological Considerations and Comparisons of Measurement Results for Extracellular Proteolytic Enzyme Activities in Seawater

**DOI:** 10.3389/fmicb.2017.01952

**Published:** 2017-10-10

**Authors:** Yumiko Obayashi, Chui Wei Bong, Satoru Suzuki

**Affiliations:** ^1^Center for Marine Environmental Studies, Ehime University, Matsuyama, Japan; ^2^Institute of Biological Sciences, Faculty of Science, University of Malaya, Kuala Lumpur, Malaysia; ^3^Institute of Ocean and Earth Sciences (IOES), University of Malaya, Kuala Lumpur, Malaysia

**Keywords:** extracellular hydrolytic enzyme, protease, activity measurement, microbial loop, organic matter degradation, low protein binding microplate, MCA substrate

## Abstract

Microbial extracellular hydrolytic enzymes that degrade organic matter in aquatic ecosystems play key roles in the biogeochemical carbon cycle. To provide linkages between hydrolytic enzyme activities and genomic or metabolomic studies in aquatic environments, reliable measurements are required for many samples at one time. Extracellular proteases are one of the most important classes of enzymes in aquatic microbial ecosystems, and protease activities in seawater are commonly measured using fluorogenic model substrates. Here, we examined several concerns for measurements of extracellular protease activities (aminopeptidases, and trypsin-type, and chymotrypsin-type activities) in seawater. Using a fluorometric microplate reader with low protein binding, 96-well microplates produced reliable enzymatic activity readings, while use of regular polystyrene microplates produced readings that showed significant underestimation, especially for trypsin-type proteases. From the results of kinetic experiments, this underestimation was thought to be attributable to the adsorption of both enzymes and substrates onto the microplate. We also examined solvent type and concentration in the working solution of oligopeptide-analog fluorogenic substrates using dimethyl sulfoxide (DMSO) and 2-methoxyethanol (MTXE). The results showed that both 2% (final concentration of solvent in the mixture of seawater sample and substrate working solution) DMSO and 2% MTXE provide similarly reliable data for most of the tested substrates, except for some substrates which did not dissolve completely in these assay conditions. Sample containers are also important to maintain the level of enzyme activity in natural seawater samples. In a small polypropylene containers (e.g., standard 50-mL centrifugal tube), protease activities in seawater sample rapidly decreased, and it caused underestimation of natural activities, especially for trypsin-type and chymotrypsin-type proteases. In conclusion, the materials and method for measurements should be carefully selected in order to accurately determine the activities of microbial extracellular hydrolytic enzymes in aquatic ecosystems; especially, low protein binding materials should be chosen to use at overall processes of the measurement.

## Introduction

In aquatic ecosystems, heterotrophic prokaryotes play important roles in organic matter cycling, including the transformation and remineralization of organic molecules and in its transfer to other organisms via trophic interactions. In order for heterotrophic bacteria that are osmotrophs to obtain nutrients from polymeric biomolecules such as proteins, these high molecular weight organic molecules must be hydrolyzed extracellularly to smaller sizes (approx. <600 Da, Nikaido and Vaara, 1985) prior to their transport across the bacterial outer membrane (Weiss et al., 1991). Thus, hydrolytic activities of extracellular enzymes in aquatic environment are investigated from the standpoint of microbial ecology, biogeochemistry, and organic geochemistry (Arnosti, 2011).

Hydrolytic enzyme activities, such as protease, glucosidase, phosphatase, and chitinase, have been detected and estimated in natural seawaters (reviewed in Hoppe et al., 2002; Arnosti, 2003) using model substrates as proxies of natural substrates. Model substrates added to the sample for measuring potential hydrolytic activities in seawater may be unlabeled oligomers (Liu et al., 2010; Liu and Liu, 2015) or labeled molecules that can be detected as hydrolytic derivatives (e.g., Arnosti, 1996; Pantoja et al., 1997; Steen et al., 2006). Among fluorogenic model substrates, which have fluorophores liberated by enzymatic hydrolysis, 4-methylumbelliferyl (MUF) substrates for α-glucosidase, β-glucosidase, and alkaline phosphatase, and 4-methylcoumaryl-7-amide (MCA) substrate for leucine-aminopeptidase are the most commonly used proxies of natural substrates for measuring individual enzymatic activities in seawater samples (Hoppe, 1993). Although, using these proxies to assess natural hydrolytic activities of enzymes in environmental samples results in some theoretical limitations and uncertainties (e.g., Steen et al., 2015), important information on biogeochemical processes in aquatic ecosystems can be obtained.

Estimating more than two hydrolytic activities in the same sample permits consideration of the nutritional mode of the bacteria and the biochemical composition of available polymeric substrates in marine systems (Nagata, 2008). For example, Fukuda et al. (2000) investigated the ratio of activities by leucine-aminopeptidase and β-glucosidase along the east-west transect of the North Pacific and suggested that there is a difference in microbial biochemical conditions between the eastern and western parts of the northern North Pacific. Sala et al. (2001) suggested that the ratio of alkaline phosphatase and aminopeptidase activities could be an indicator of nitrogen and phosphate limitation in the microbial community.

Enzyme activity in bulk seawater samples are often operationally divided into fractions such as “particle-associated” and “dissolved (cell-free)” activities by taking measurements separately on seawater that passes through filters of a specified pore size, as well as unfiltered samples. These fractionations may provide insights into the natural forms of hydrolytic enzymes in seawater and the ecological roles that each play (Arnosti, 2003). Smith et al. (1992) and Karner and Herndl (1992) showed that particle (marine snow)-associated hydrolytic activities were much higher than those in the surrounding seawater at least for their tested enzyme types. Meanwhile, dissolved (free) enzymes have been reported to make substantial contributions to the total activity (e.g., Keith and Arnosti, 2001; Obayashi and Suzuki, 2008a; Baltar et al., 2016), although their ratios vary depending on the sampling conditions and enzyme type.

Proteins and peptides should be good nutrition for heterotrophic prokaryotes after suitable hydrolysis by extracellular enzymes, and thus extracellular proteases are one of the most important hydrolytic enzymes in aquatic microbial ecosystems. Measurements of extracellular proteolytic enzyme activity in aquatic samples using fluorogenic model substrates have been popular since their introduction (e.g., Hoppe, 1993), especially for leucine aminopeptidase, because of their high sensitivity and easiness of the method. Not only aminopeptidase activity but a number of diverse proteolytic enzymes and the importance of trypsin-type endopeptidases, which cleave peptide bonds within a peptide, in natural seawater were also reported using 16 different MCA substrates (Obayashi and Suzuki, 2005). To discuss the relationships or interactions with physical and chemical environmental conditions, or to link those with genomic or metabolomic information on microbial communities, high-resolution reliable measurement data on extracellular proteolytic enzyme activities are required. High-resolution analysis requires many samples to be measured at one time, such as collecting samples at many depth layers at each sampling site, many size fractionations, or testing with different kinds of model substrates. To get reliable activity data, many samples should be analyzed as soon as possible after sampling (German et al., 2011) and each sample measurement should be performed in replicate. Moreover, for reliable estimation of the potential activity of microbial extracellular hydrolytic enzymes in the aquatic environment, many factors should be considered. For example, Obayashi and Suzuki (2008b) pointed out that adsorption effects to the filter for size fractionation could lead underestimation of the enzyme activity in the filtrates depending on the type of filter material and that the effects were different among the type of enzymes. Recently, not only a standard spectrofluorometer with a cuvette, a fluorometric microplate reader with a micro-well plate has been also applied as a device to read fluorescent intensity during a measurement of activity (e.g., Baltar et al., 2010). Microplate reader offers considerable advantage to get measurement data for many samples at one time, however, using 96-well microplates, water sample volume is small and the ratio of the area touching plate material (the wall and bottom of each well) to the sample volume is relatively large. Considering a kind of adsorption effect likely to filters for size fractionation, samples in a microplate might be more susceptible to some kinds of artifact than in a larger volume cuvette. However, many researches might be performed with microplate not always taking care about the types of microplate materials, and to our knowledge, systematic comparison between data obtained from microplates and cuvettes has not been reported so far regarding the measurement of extracellular enzyme activity in a natural seawater sample. Here, we examine several concerns regarding to achieve high-resolution reliable estimations of extracellular proteolytic enzyme activities in seawater using many kinds of MCA substrates and a fluorometric microplate reader.

## Materials and methods

### Seawater samples

Seawater samples for each test were collected by bucket or Van Dorn water sampler along the coast of Ehime Prefecture, Japan, and filtered through nylon mesh (150 or 50 μm) into bottles to remove large particles. Polycarbonate 500 mL bottles were used as a sample container, except for the experiment to compare several types of containers. Samples were immediately placed on ice for transport to the laboratory where the samples were refrigerated at 4°C until proceeding with the assays within several hours. In general, to assess the extracellular enzyme activities in natural aquatic samples the activities should be measured as soon as possible after sampling, to minimize the possible alteration after sampling such as degradation of dissolved enzymes and changing of microbial community and their activity. Even though the main purpose of each experiment in this study was to compare the data for the same water sample using different methodological conditions, we conducted each experiment as soon as possible after seawater sampling (within several hours), except for the preservation test in different sample containers. When we kept water samples for a short time before measurement, samples were kept cool and in the dark for the least degradation of enzymes possible, although there were still unavoidable possibilities of microbial cell lysis at 4°C.

For some experiments, an aliquot of sample was filtered through a 0.2 μm pore size polycarbonate Nuclepore filter (Whatman), then both unfiltered and filtered (<0.2 μm) seawater samples were used.

### Enzyme activities measurement

The potential activities of extracellular proteolytic enzymes in seawater samples were measured using 17 MCA substrates (Table 1, Peptide Institute): 5 for aminopeptidase, 10 for trypsin, and 2 for chymotrypsin. Enzyme activities measurement was conducted as follows with modifications for the different methods tested, as noted below.

**Table 1:** List of fluorogenic substrates used in the present study.

**Substrate**		**Experiment**						
**Name**	**Substrate for**	**Method comparison**	**Kinetics**	**Microplate comparison**	**Solvent**			**Sample container**
		**Methods A,B,C**	**Methods B,C**	**3 Suppliers**	**DMSO 10% vs. 2%**	**DMSO 2% vs. 1%**	**DMSO 2% vs. MTXE 2%**	
Arg-MCA	Aminopeptidase	+		+		^*^	^*^	
Leu-MCA	Aminopeptidase	+	+	+	+	+	+	+
Ala-MCA	Aminopeptidase	+	+	+	+	+	+	+
Lys-MCA	Aminopeptidase	+		+		+	+	
Phe-MCA	Aminopeptidase	+		+		+	+	
Bz-Arg-MCA	Trypsin	+		+		+	+	
Z-Phe-Arg-MCA	Trypsin	+				^*^	^*^	
Glt-Gly-Arg-MCA	Trypsin	+		+		+	+	
Boc-Leu-Gly-Arg-MCA	Trypsin	+		+		+	+	
Boc-Leu-Thr-Arg-MCA	Trypsin	+		+		+	+	
Boc-Phe-Ser-Arg-MCA	Trypsin	+	+	+	+	^*^	+	+
Boc-Val-Pro-Arg-MCA	Trypsin	+		+		+	+	
Boc-Leu-Ser-Thr-Arg-MCA	Trypsin	+	+	+	+	+	+	+
Boc-Val-Leu-Lys-MCA	Trypsin	+		+		+	+	
Boc-Glu-Lys-Lys-MCA	Trypsin	+		+		+	+	
Suc-Ala-Ala-Pro-Phe-MCA	Chymotrypsin	+	+	+	+	+	+	+
Suc-Leu-Leu-Val-Tyr-MCA	Chymotrypsin	+			^*^	^*^	^*^	
MUF-phosphate	Phosphatase		+					

+Substrate used in experiment;

* *Substrate not soluble in one or both concentrations of solvent. MCA, 4-methylcoumaryl-7-amide; Bz, Benzoyl; Z, Carbobenzoxy; Boc, t-Butyloxycarbonyl; Suc, Succinyl; MUF, 4-Methylumbelliferyl*.

MCA substrates were dissolved in solvents dimethyl sulfoxide (DMSO) or 2-methoxyethanol (MTXE) to prepare stock solutions (10 or 20 mM). For assay, 10× substrate working solutions were prepared from the stock solutions in autoclaved artificial seawater and solvent to control for solvent concentration in the solution, with substrate and solvent concentrations 10 times higher than the target final concentrations in assay. Seawater samples and 10× substrate solutions were mixed in disposable cuvettes or 96-well microplates and incubated at 25°C in the dark to measure potential enzyme activities. The fluorescence of the hydrolytic product, 7-amino-4-methylcoumarin (AMC), was measured several times at intervals (t0, t1, t2, t3; typically 1 h interval) during the incubation. The excitation/emission wavelengths for fluorescence measurements were 380/460 nm on a spectrofluorometer (Hitachi F-2500) or 380/440 nm on a microplate reader (Corona SH8100Lab). A solvent blank (seawater sample with solvent but without substrate) was also prepared and subjected to fluorescence measurements along with samples. After subtracting the solvent fluorescence blank, the concentration of AMC generated during the incubation was calculated using a calibration curve prepared by measuring fluorescence intensity of AMC solutions at seven concentrations (0–1 μM) under the same conditions as the sample measurements. To measure the non-enzymatic produced AMC during incubation, collected seawater was autoclaved and prepared and assayed as an inactivated control under the same conditions as the intact sample. The hydrolysis rate of the substrate in the seawater sample, namely, extracellular enzyme activity in seawater, was calculated by determining the increase in AMC concentration with time after subtracting the concentration of non-enzymatic produced AMC estimated in autoclaved seawater.

Every assay was performed in triplicate using three cuvettes or three wells in a microplate for each sample and substrate pair.

### Comparison methods for protease activities measurement

Extracellular proteolytic enzyme activities in natural seawater samples were measured using different assay methods (Methods A, B, and C) simultaneously with the same working solutions. In Method A, we used a spectrofluorometer with disposable cuvettes, while Methods B and C were conducted on a fluorometric microplate reader with regular and low protein binding microplates, respectively. The excitation/emission wavelengths recommended by the supplier of MCA substrates (Peptide Institute) for the assay were 380/460 nm; however, to obtain a higher intensity fluorescence signal from smaller sample volumes used on microplates (Methods B and C), we set the emission wavelength at 440 nm, the wavelength of maximum fluorescence intensity for AMC. We confirmed that readings taken at 440 and 460 nm provided equivalent results when the corresponding calibration curve was applied. Following are brief overviews of Methods A, B, and C:

Method A (cuvette) was conducted with a reaction volume of 1 mL in a disposable cuvette made from polymethylmethacrylate (PMMA). Fluorescence was measured by a spectrofluorometer at excitation/emission wavelengths of 380/460 nm.

Method B (regular microplate) was conducted with a reaction volume of 300 μL in regular 96-well black microplates made from polystyrene (Nunc #237107). Fluorescence was measured by a microplate reader in fluorescence mode at an excitation/emission wavelength of 380/440 nm.

Method C (low protein binding microplate) was conducted as for Method B except with a low protein binding, black, 96-well microplate made from polystyrene coated with methacryloyloxyethyl phosphorylcholine (MPC) (Nunc #245393).

Pearson's correlation coefficient and simple regression were used to compare data obtained from different methods in pairwise comparisons (Methods A vs. B, Methods A vs. C, Methods C vs. B). Regression analyses of pairs of methods were performed with combined dataset from unfiltered and filtered seawater samples for “all estimated activities” and for the “aminopeptidase activities,” “trypsin-type activities,” and “chymotrypsin-type activities.”

### Kinetic experiments

Using selected MCA substrates for proteases (Table 1), kinetic experiments were performed, and the Michaelis plots obtained by Method B (regular microplate) and Method C (low protein binding microplate) were compared. For the kinetic experiments, MCA substrates were added to samples at final concentrations of 0, 10, 20, 50, 100, 150, 200, and 250 μM. Hydrolysis rates of substrates were measured by Methods B and C with the protocol given above.

To determine whether differences in results between Methods B and C are proteolytic enzyme-specific, the same experiment was conducted using MUF substrate for phosphatase (MUF-phosphate, Wako Chemicals) at a final concentration of 0, 10, 30, 60, 100, 160, and 200 μM. Measurement of the hydrolysis rate of the MUF substrates was conducted by the same method as for the MCA substrates, except that the excitation/emission wavelengths of 365/445 nm were used to detect the 4-methylumbelliferon product.

Based on the measured hydrolytic activities, *V*, and the substrate concentrations, [*S*], the theoretical maximum activity, *V*_*max*_, and the Michaelis constant, *K*_*m*_, were estimated by curve fitting using software OriginPro 9.1 to the Michaelis–Menten equation:

(1) $$\begin{array}{r} V=V_{\max}\times \left[S\right]/\left(K_{m}+\left[S\right]\right) \end{array}$$

### Comparison of low protein binding microplates from different suppliers

Low protein binding 96-well black microplates from different suppliers were tested using seawater collected by bucket and strained through 50 μm nylon mesh into polycarbonate bottles. An aliquot of seawater (180 μL) was mixed with 20 μL of each of 15 MCA substrate solutions (Table 1; final concentration, 200 μM substrate, 2% DMSO) in three different microplates. Nunc low protein binding plate (Nunc #245393, as described above), Greiner Bio-one No-binding plate (Greiner 655900), and SUMILON Proteosave plate (Sumitomo Bakelite MS-8296K) were used to measure proteases activities in the same seawater sample with the same substrate solution, and the results were compared. Enzyme activities were measured using the protocol described above.

### Examination of solvent

The manufacturer of the MCA substrates (Peptide Institute) recommends using DMSO to prepare stock solutions. Considering the possibility of solvent bias in activities measurement, a lower concentration of solvent in assay is better. However, lower solvent concentration may result in reduced solubility of the substrate in seawater. To examine the effect of DMSO, two experiments were conducted with different DMSO concentrations: (1) kinetic experiments comparing 10% (v/v) and 2% (v/v) DMSO, and (2) comparison of the activities measurement with 2 and 1% DMSO with 200 μM substrate final concentration.

Substrate working solution (10×) were prepared for each substrate concentration with 100% DMSO for final 10% DMSO in the assay, while those were prepared with autoclaved artificial seawater containing 20% DMSO for final 2% in the assay. Using low protein binding, 96-well microplates (Nunc), natural seawater sample 270 μL and 10× substrate working solution 30 μL were mixed in each well of the microplate. For this experiment, five MCA substrates (2 for aminopeptidase, 2 for trypsin, and 1 for chymotrypsin) were used (Table 1). Calibration curves of AMC were generated for each assays conducted with 10 and 2% DMSO.

Activity measurements were compared between 2 and 1% DMSO concentrations with 200 μM final substrate concentration. For this experiment, 15 substrates (Table 1) and low protein binding microplates from three suppliers (Nunc, Greiner, Sumitomo, as described above) were used. The seawater sample (180 μL) and 20 μL of 2 mM substrate working solution with 20 or 10% DMSO were mixed in each well of the microplate. Calibration curves of AMC were generated for each the assays conducted with 2 and 1% DMSO.

Previous studies used 2-methoxyethanol (MTXE, methylcellosolve) as a solvent of substrates to measure potential activities of extracellular hydrolytic enzymes in seawater (e.g., Fukuda et al., 2000). We also tested MTXE instead of DMSO as a solvent to dissolve the MCA substrates for protease assay. Substrate stock solutions (20 mM) were prepared with MTXE, and working solutions (10×) of each substrate, which contain 2 mM substrate and 20% MTXE, were prepared from the stock solution and autoclaved artificial seawater. A calibration curve of AMC with 2% MTXE was also prepared, and other procedures for fluorescence measurement and activities estimations were conducted as described above.

Significance of differences between 1 and 2% DMSO concentrations and different solvents (2% MTXE and 2% DMSO) were tested by Student's *t*-test.

### Sample containers for seawater collection

To test the effect of sampling container material, the following five types of containers were used: 500 mL polycarbonate bottles (PC500) (Nalgene), 50 mL polypropylene tubes supplied by Corning (PPC) and Eppendorf (PPE), 50 mL polyethylene terephthalate tubes (PET) (Corning), and low protein binding ProteosaveSS 50 mL tubes (SS) (Sumitomo Bakelite Co., Ltd.). The four 50 mL tubes had a similar shape of ordinary plastic centrifugal tubes. Natural seawater samples were collected by a bucket and immediately transferred to these containers through 50 μm nylon mesh. All seawater samples were kept cool until measurements of proteases activities. Proteases activities were measured using five MCA substrates (2 for aminopeptidase, 2 for trypsin, and 1 for chymotrypsin) at several hours after sampling, and at 1 day (26 h) and 2 days (47 h) after sampling. Reaction volume for the assay was 200 μL (180 μL seawater sample + 20 μL substrate solution) in low protein binding microplate (Nunc), and the final concentrations in the mixture were 200 μM substrate with 2% DMSO. Other procedures for fluorescence measurement and enzyme activities estimation were the same as described above.

## Results and discussion

### Microplates for enzyme activities measurement in seawater

#### Comparison of activity estimation by methods A (cuvette), B (regular microplate), and C (low protein binding microplate)

**Figure 1:**
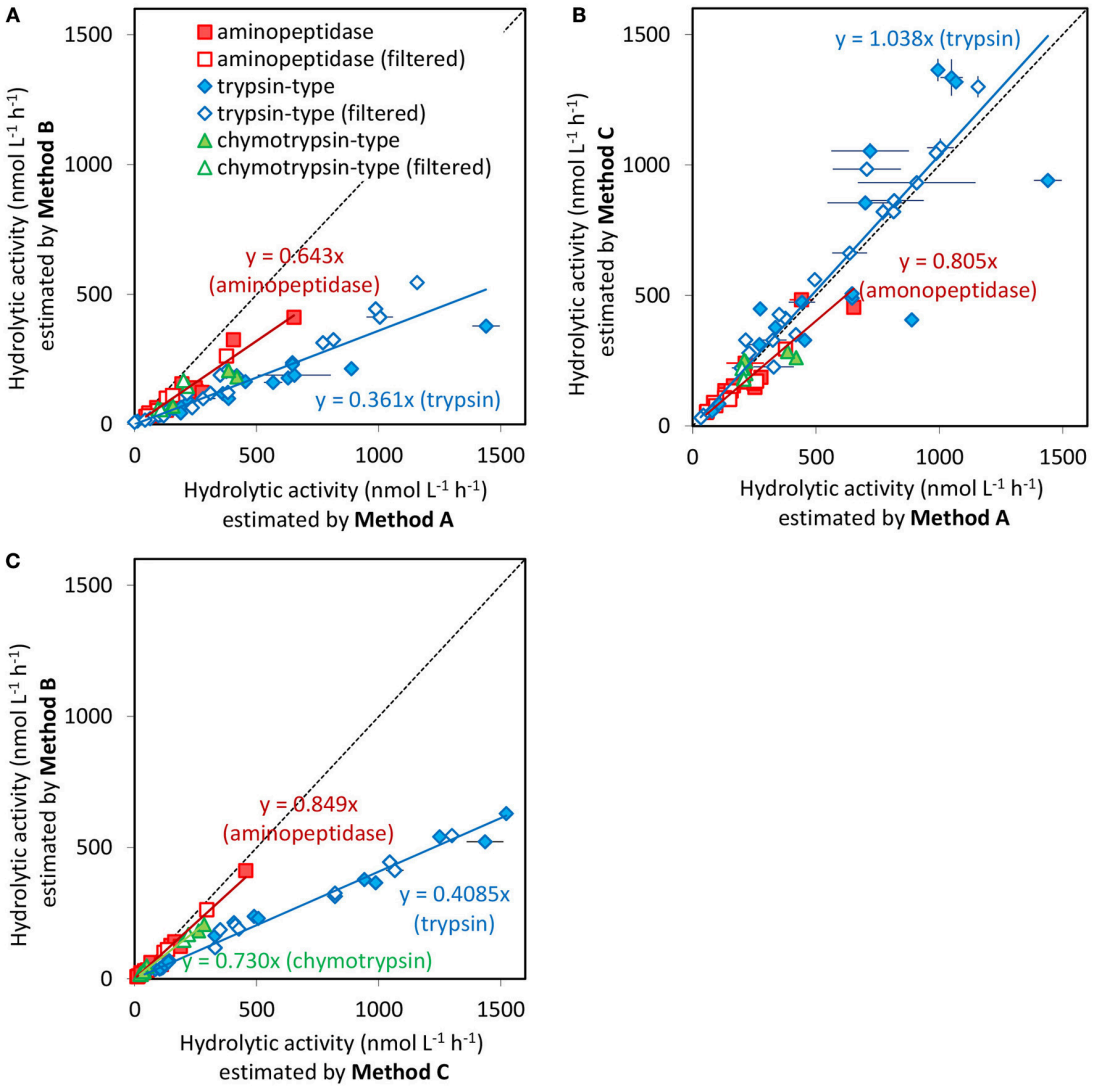
Scatter plots of hydrolytic activities measured by **(A)** Method A (spectrofluorometer with disposable cuvettes) and Method B (fluorometric microplate reader with regular polystyrene microplate), **(B)** Method A and Method C (fluorometric microplate reader with low protein binding microplate), and **(C)** Method C and Method B. Activities measured for all samples by each of the two methods are plotted. The shapes of symbols indicate enzymes type, and filled and opened symbols indicate unfiltered seawater samples and <0.2 μm filtered seawater (dissolved fraction), respectively. Samples from different dates are not differentiated. Error bars are the standard deviations of triplicate sample preparations. Regression lines for aminopeptidase and trypsin-type endopeptidase and their equations are also shown. These regression analyses were performed with combined dataset from unfiltered and filtered samples. Dashed line indicates 1:1.

Figure 1 shows the relationship hydrolytic activities measurements obtained by each method for the same samples. Data from unfiltered and 0.2 μm filtered seawater samples were indicated as filled and opened symbols, respectively. Different shapes of the symbols in Figure 1 refer to different types of enzyme activities estimated by using different substrates.

The protease activities measured by Method B (microplate reader with regular polystyrene microplates) were systematically lower than those by Method A (spectrofluorometer with cuvette; Figure 1A) and Method C (microplate reader with low protein binding microplates; Figure 1C). For the comparison of Method B to Method A, the linear relationship between measured activity for the two methods on all samples was significant [*n* = 64 (unfiltered and filtered samples were combined), *r* = 0.953, *p* < 0.0001] with a regression coefficient of 0.394 ± 0.015 (regression line for all data not shown in Figure 1A) indicated that the measured values obtained by Method B were only about 39% of those measured by Method A. The linear relationships between the results of Methods B and A were significant for both aminopeptidase activity (*n* = 20, *r* = 0.988, *p* < 0.0001) and trypsin-type activity (*n* = 36, *r* = 0.972, *p* < 0.0001). Regression line was not shown for chymotrypsin-type activity in Figure 1A because of the limited number of data points comparing with aminopeptidase and trypsin-type activity. The regression coefficient for trypsin-type activity (0.361 ± 0.014) was significantly smaller (*t* = 4.12, *p* < 0.0005) than that for aminopeptidase activity (0.643 ± 0.023). Although, there is not absolute evidence that higher estimation is more accurate, these results seem to suggest that Method B (microplate reader with regular polystyrene microplates) resulted in an underestimation of extracellular enzyme activity in natural seawater, and this effect was greater for trypsin-type activity than for aminopeptidase activity. Similarly, for the comparison of activities measurements by Method B and Method C (Figure 1C) shows that aminopeptidase activity and trypsin-type activities measured by Method B are 85 and 41%, respectively, of the measured values by Method C. For this comparison, the analytical methods of Methods B and C are identical and the differences in measurement are attributable to the material of the microplates.

The linear relationship between the enzyme activities measurements obtained by Methods A and C was significant (*n* = 64, *r* = 0.968, *p* < 0.0001) and the regression coefficient was near 1 (1.015 ± 0.031), indicating that proteases activities measurements by these two methods are equivalent. Separate analyses for each of the enzyme types also were significant with regression coefficients for aminopeptidase and trypsin-type activity of 0.805 ± 0.039 and 1.038 ± 0.044, respectively (Figure 1B); the difference between these two coefficients was not significant (*t* = 0.95, *p* > 0.35). These results indicated that the systematic underestimation of enzyme activities using Method B can be avoided by using low protein binding microplates.

#### Factors causing reduced enzyme activities measurements with regular polystyrene microplates

To clarify the discrepancies between the results obtained using regular microplates and low protein binding microplates, kinetic experiments were conducted based on the following hypotheses. If the observed underestimation is due solely to the adsorption of the artificial substrate and not due to the adsorption and deactivation of enzymes onto the surface of the polystyrene microplates, the measured activity by Method B should become saturated at higher level of the substrate than by Method C, and the differences between the measurements by Methods B and C should diminish at higher concentrations of substrate. On the other hand, if the reduced measurements are due to adsorption/deactivation of enzymes, the activities measurement by Method B should become saturated at almost the same level of substrate concentration as for Method C; namely, the Michaelis constant, *K*_*m*_, should be at a similar value, and the maximum activity, *V*_*max*_, estimated by Method B should be smaller than that for Method C.

**Figure 2:**
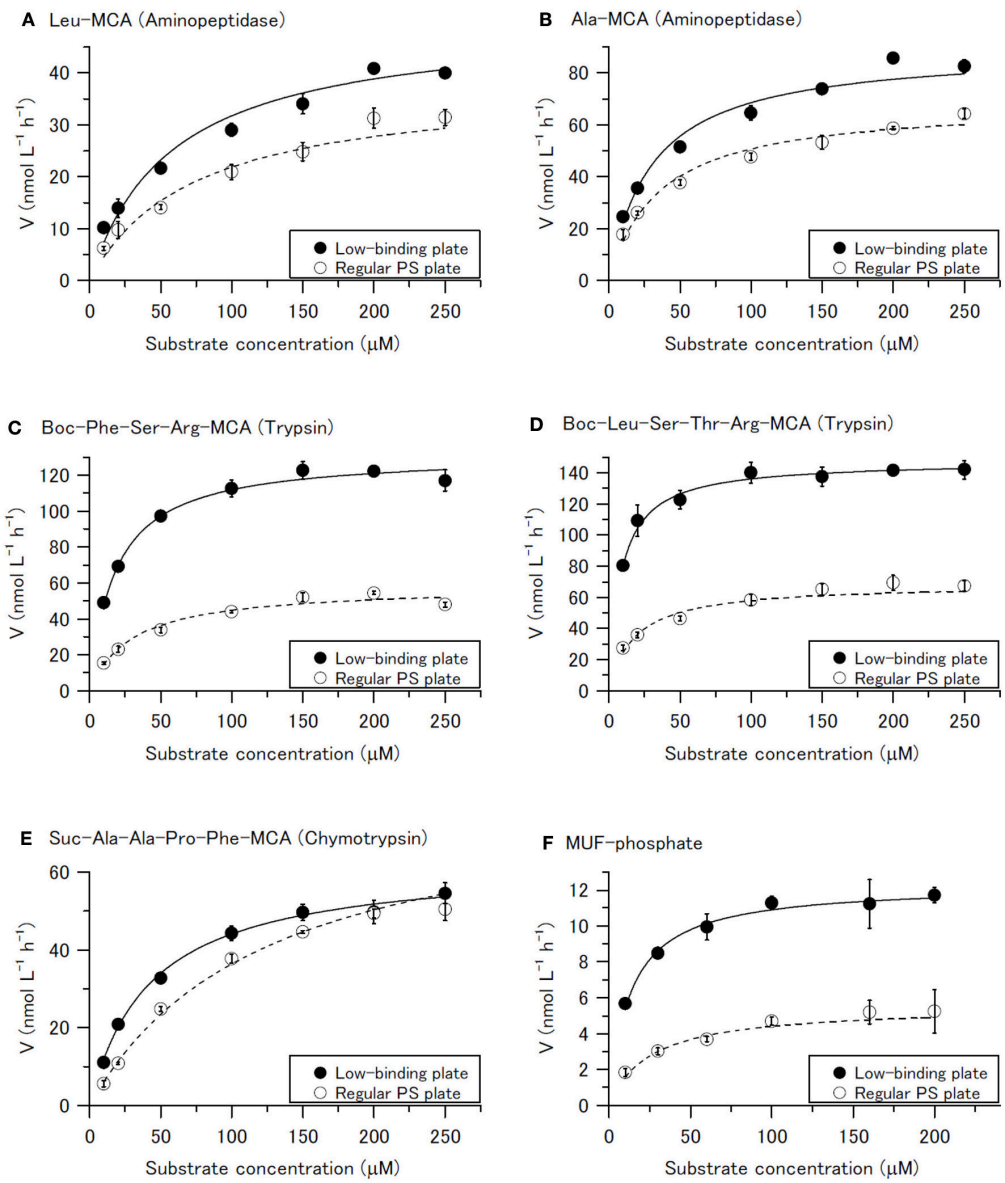
Michaelis plot of hydrolysis of **(A)** Leu-MCA (substrate for aminopeptidase), **(B)** Ala-MCA (substrate for aminopeptidase), **(C)** Boc-Phe-Ser-Arg-MCA (substrate for trypsin), **(D)** Boc-Leu-Ser-Thr-Arg-MCA (substrate for trypsin), **(E)** Suc-Ala-Ala-Pro-Phe-MCA (substrate for chymotrypsin), and **(F)** MUF-phosphate (substrate for phosphatase) measured by Method C (low protein binding microplate) and Method B (regular polystyrene microplate). Error bars are the standard deviation of triplicate sample preparations (each sample and substrate pair was incubated and measured in 3 wells separately). Solid lines and dashed lines indicate curves fitting data from the low protein binding microplates and regular polystyrene microplates, respectively, to the Michaelis-Menten equation.

Our results show that aminopeptidase and trypsin-type activities estimated by Method B were lower than those by Method C even at higher substrate concentrations (Figure 2), although not much differences in chymotrypsin-type activity at the highest substrate concentration for Methods B and C. By curve fitting to the Michaelis–Menten equation, *V*_*max*_ of aminopeptidase by Methods B and C was estimated to be 38.0 ± 5.6 and 50.1 ± 3.9 nmol L^−1^ h^−1^, respectively, for hydrolysis of Leu-MCA and 68.7 ± 3.0 and 89.3 ± 2.0 nmol L^−1^ h^−1^, respectively, for Ala-MCA. *V*_*max*_ estimation of trypsin-type activity by Methods B and C was 58.2 ± 1.9 and 131.8 ± 4.6 nmol L^−1^ h^−1^, respectively, for hydrolysis of Boc-Phe-Ser-Arg-MCA and 68.3 ± 3.0 and 147.4 ± 2.5 nmol L^−1^ h^−1^, respectively, for hydrolysis of Boc-Leu-Ser-Thr-Arg-MCA. Thus, for aminopeptidase (Figures 2A,B) and trypsin-type activity (Figures 2C,D), *V*_*max*_ by Method B were 76–77% and 44–46% of those by Method C, respectively. These results indicate that significant adsorption of enzyme itself onto the surface of the regular polystyrene microplate occurred and that it could result in the underestimation of protease activity in seawater, especially for trypsin-type enzymes.

If the reason for lower enzyme activity is due solely to adsorption of the enzyme, the Michaelis constant (half-saturation constant) should be the same for a sample by both assay methods. However, the *K*_*m*_ estimated by curve fitting the trypsin-type activity data obtained by Methods B and C to the Michaelis–Menten equation were different: 30.1 ± 3.8 and 17.9 ± 1.6 μM, respectively, for hydrolysis of Boc-Phe-Ser-Arg-MCA and 18.1 ± 2.6 and 8.4 ± 0.4 μM, respectively, for hydrolysis of Boc-Leu-Ser-Thr-Arg-MCA. These differences could be explained by the adsorption of the substrates onto the regular polystyrene microplates and the available substrate concentration becoming lower than expected.

We also conducted the same kinetic experiment for phosphatase activity in terms of hydrolysis of MUF-phosphate to determine whether the discrepancy in measurement results between Methods B and C was specific to proteolytic enzymes, which is estimated by hydrolysis of oligopeptide analog MCA substrates. The results of phosphatase activity assays were almost the same as those of trypsin-type proteases activity tests with Boc-Phe-Ser-Arg-MCA and Boc-Leu-Ser-Thr-Arg-MCA. The phosphatase activity, *V*_*max*_ of hydrolysis of MUF-phosphate, was 5.5 ± 0.4 nmol L^−1^ h^−1^ by Method B, which is 45% of that measured by Method C (12.3 ± 0.4 nmol L^−1^ h^−1^) for the same seawater sample (Figure 2F). The *K*_*m*_ estimated by curve fitting to the data obtained from a regular plate and a low protein binding plate was 25.2 ± 6.2 and 12.8 ± 1.3 μM, respectively.

From these results, we conclude that the reduced enzyme activities measurements with the use of regular polystyrene microplates (Method B) are attributable to the adsorption of both enzymes and substrates to the microplate surface. Although, the relationship between enzyme adsorption (binding) onto the polystyrene surface and deactivation of the adsorbed enzymes was not determined, Calliou et al. (2008) reported that adsorbed enzymes could be deactivated based on their model experiments. Among the proteases tested in our study, adsorption effects appeared to be more severe for trypsin-type enzymes than for aminopeptidases. A similar suppression of enzymatic activities measurements in seawater was previously reported during filtration (Obayashi and Suzuki, 2008b). In that case, trypsin-type enzyme appeared to be much more readily adsorbed onto the mixed cellulose esters filter (0.22 μm pore size) than aminopeptidase; as a result, not only particles in the sample but also much of the dissolved trypsin were removed by filtration. Taking the results of previous and present studies together, we suppose that trypsin-type enzymes in seawater are more easily adsorbed on some kinds of solid surfaces and/or deactivated on solid surfaces than are aminopeptidases. These results imply that enzyme behaviors and characteristics in natural environment are different for extracellular aminopeptidases and trypsin-type endopeptidases.

#### Low protein binding 96-well microplates

**Figure 3:**
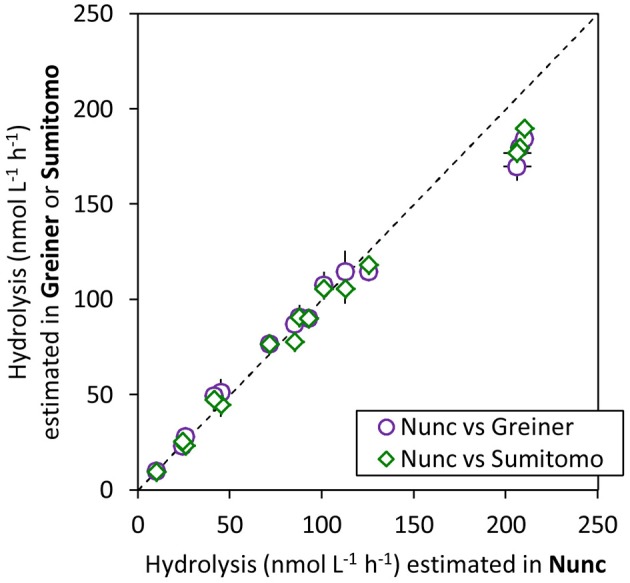
Comparison between hydrolytic activities measured in Greiner Bio-one No-binding microplate (Greiner) and SUMILON Proteosave microplate (Sumitomo) to Nunc low binding microplate (Nunc), for the same seawater samples, using the same analytical method except for the microplates. Error bars are the standard deviation of triplicate sample preparations (same sample incubated in 3 wells). Dashed line indicates 1:1.

Figure 3 shows a comparison of estimated proteases activities in the same seawater sample using low protein binding, black, 96-well microplates from three suppliers. All three low protein binding microplates tested here gave similar measurements of hydrolytic activities of all tested substrates. Although, most experiments in present study were conducted using Nunc low binding microplates, Greiner Bio-one No-binding black 96-well microplates and SUMILON ProteosaveSS black microplates can provide equivalent results.

### Solvent for substrates

#### DMSO concentration in the assay

Most MCA substrates need to be dissolved in organic solvent prior to mixing with the seawater sample. DMSO is a good solvent for dissolving MCA substrates; however, toxic effects to microbial cells in seawater and other unexpected effects could occur during the incubation and affect the measurement of extracellular enzyme activities in seawater samples. To minimize these types of artifacts, a lower concentration of organic solvent in the assay mixture is preferred. However, too low of a solvent concentration with a high concentration of substrate might result in solubility difficulties during the assay.

**Figure 4:**
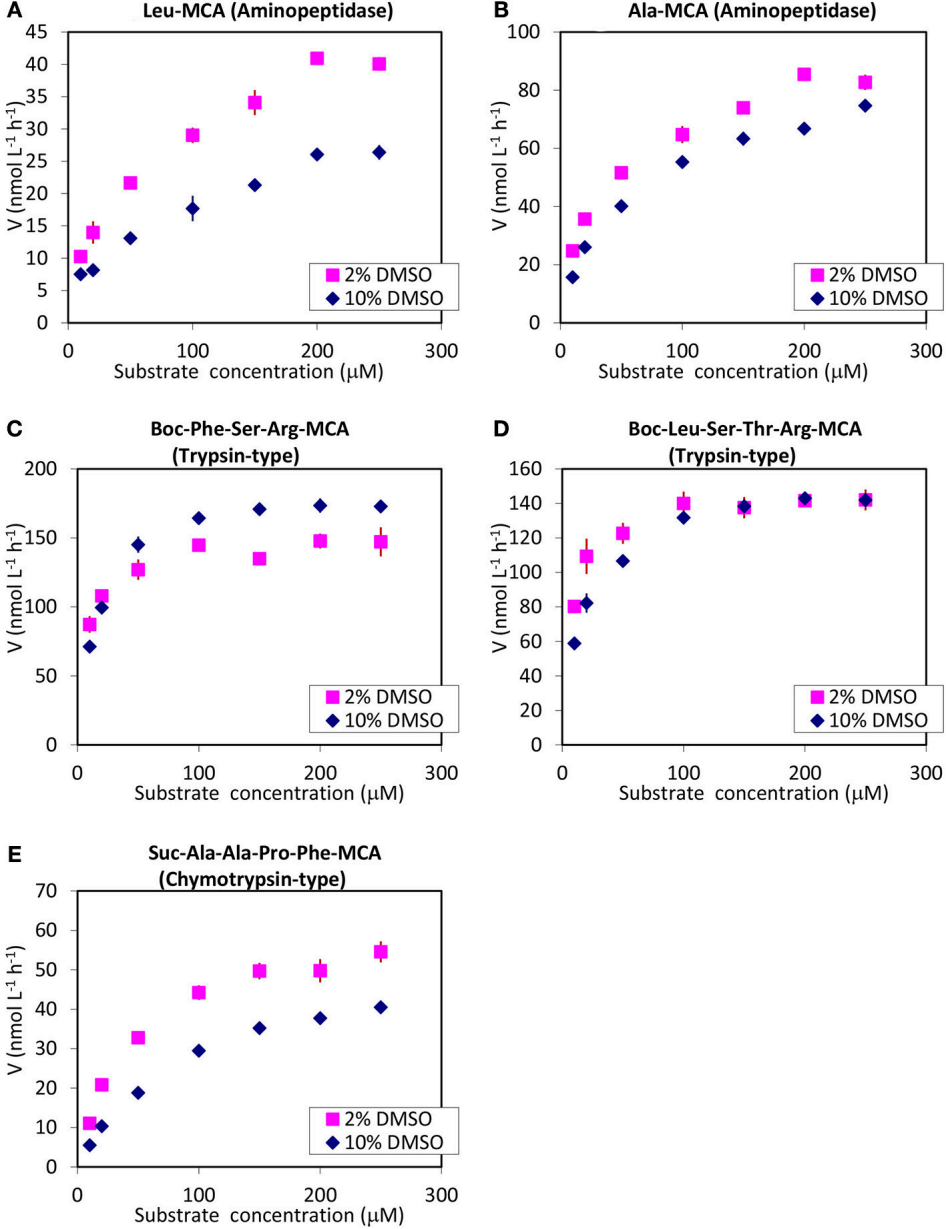
Michaelis plots of the hydrolysis of **(A)** Leu-MCA (substrate for aminopeptidase), **(B)** Ala-MCA (substrate for aminopeptidase), **(C)** Boc-Phe-Ser-Arg-MCA (substrate for trypsin), **(D)** Boc-Leu-Ser-Thr-Arg-MCA (substrate for trypsin), and **(E)** Suc-Ala-Ala-Pro-Phe-MCA (substrate for chymotrypsin), measured in 2 and 10% DMSO. Error bars are the standard deviation of triplicate sample preparations (same sample incubated in 3 wells).

Michaelis plots for assays conducted with 10 and 2% DMSO with various substrate concentrations of five selected substrates (2 for aminopeptidase, 2 for trypsin, and 1 for chymotrypsin) are shown in Figure 4. Aminopeptidase and chymotrypsin-type activities were estimated lower in 10% DMSO than in 2% DMSO, irrespective of substrate type. In the case of the trypsin-type activity, hydrolysis rates in 2% DMSO were higher than in 10% DMSO for lower concentration of substrate, while rates of both were at the same level (hydrolysis of Boc-Leu-Ser-Thr-Arg-MCA, Figure 4D), or the rate in 2% DMSO was lower than that in 10% DMSO condition (hydrolysis of Boc-Phe-Ser-Arg-MCA, Figure 4C) at higher concentration of substrate. Previous studies have reported that a large proportion of aminopeptidase activity in seawater was detected from the bacterial cell size fraction as ectoenzymes, while trypsin-type activities were mostly detected in the dissolved (<0.2 μm filtered) fraction (Karner and Rassoulzadegan, 1995; Hoppe et al., 2002; Obayashi and Suzuki, 2008a; Bong et al., 2013). Higher contributions of bacterial cell-associated fractions of chymotrypsin-type activity were reported in some cases (Bong et al., 2013). The difference in the apparent DMSO effects between trypsin-type and other enzymes could be due to predominant state of existence in seawater: Lower activity of aminopeptidase and chymotrypsin-type enzymes in 10% DMSO in this study might reflect the toxic effects of DMSO to microbial cells during the incubation. Taking the Michaelis plot for the assay with Boc-Leu-Ser-Thr-Arg-MCA as a substrate (Figure 4D), 10% DMSO appears to act as a competitive inhibitor for dissolved trypsin-type enzyme. The reason for the discrepancy between the results of hydrolysis of two substrates for trypsin (Boc-Phe-Ser-Arg-MCA and Boc-Leu-Ser-Thr-Arg-MCA) at higher concentration of substrates was not clear; however, it might be related to the solubility of Boc-Phe-Ser-Arg-MCA as explored below.

Among the 17 tested MCA substrates listed in Table 1, Z-Phe-Arg-MCA (substrate for trypsin) and Suc-Leu-Leu-Val-Tyr-MCA (substrate for chymotrypsin) were not soluble enough to use with 2% DMSO, and additionally, Arg-MCA (substrate for aminopeptidase), and Boc-Phe-Ser-Arg-MCA (substrate for trypsin) could not be used with 1% DMSO. These substrates produced visible precipitates or aggregates in the 10× working solution or upon mixing with seawater samples for assay.

**Figure 5:**
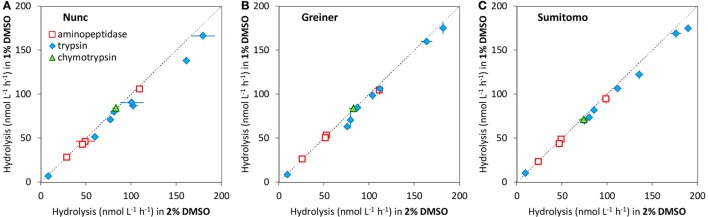
Scatter plots of hydrolytic activities measured in 2 and 1% DMSO on **(A)** Nunc low binding microplate, **(B)** Greiner Bio-one No-binding microplate, and **(C)** SUMILON Proteosave microplate. Error bars are the standard deviation of triplicate sample preparations (same sample incubated in 3 wells). Dashed line indicates 1:1.

Excluding these four substrates, which were not sufficiently soluble, we compared the enzyme activities measurements in assays with 2 and 1% DMSO (Figure 5). For this test, the final concentration of each substrate was set to 200 μM, which is the saturation level for most of the tested substrates. Hydrolytic activity results for 2 and 1% DMSO were similar and the differences were not significant (*p* = 0.295 for all pairs, *n* = 39; *p* = 0.274 for aminopeptidase, *n* = 12; *p* = 0.256 for trypsin-type, *n* = 24; *p* = 0.965 for chymotrypsin-type, *n* = 3). Steen et al. (2015) examined the effect of DMSO on aminopeptidase (Leu-MCA hydrolysis) kinetics in river water samples and reported that DMSO at 4% or less did not influence the estimation of *K*_*m*_ but use of 5% DMSO produced different results.

Although, the actual effects of DMSO might differ among the enzymes, estimation of hydrolysis for many MCA substrates with 2% DMSO seemed to provide reliable estimation of protease activity in environmental seawater samples. For substrates with high solubility throughout the assay, a lower percentage of DMSO should be acceptable for measuring enzyme activity in seawater samples.

#### Comparison of using 2% DMSO and 2% MTXE in assay

**Figure 6:**
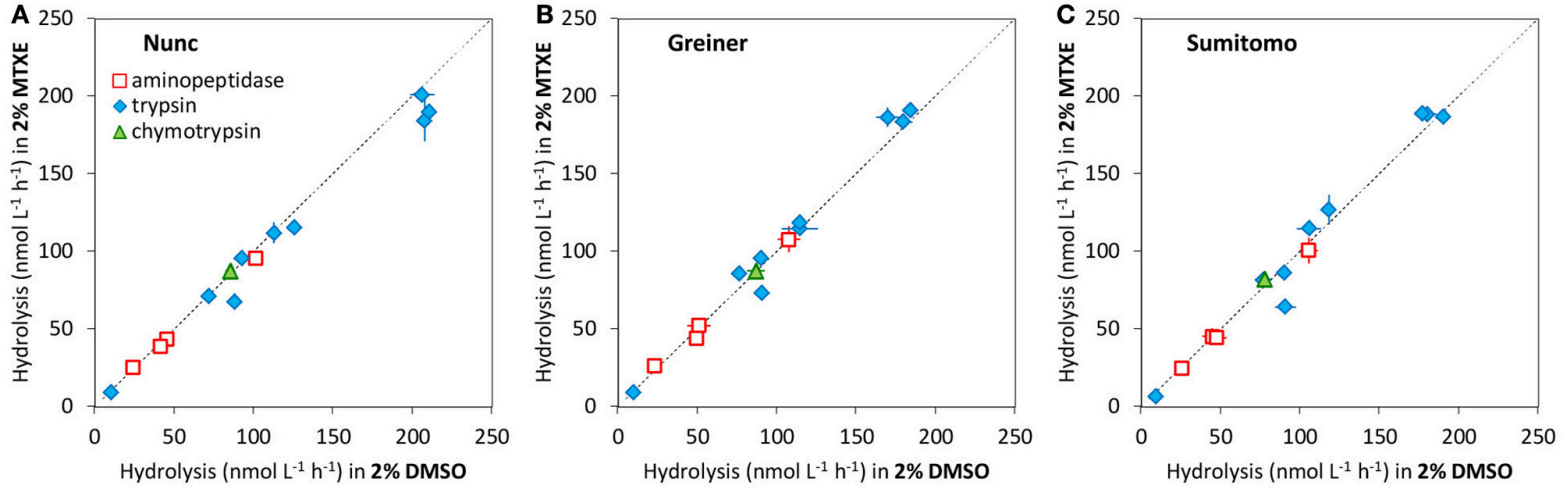
Scatter plots of hydrolytic activities measured in 2% DMSO and 2% MTXE on **(A)** Nunc low binding microplate, **(B)** Greiner Bio-one No-binding microplate, and **(C)** SUMILON Proteosave microplate. Error bars are the standard deviation of triplicate sample preparations (same sample incubated in 3 wells). Dashed line indicates 1:1.

In previous studies, MTXE was used to prepare stock solutions of hydrolytic substrates. Here, we compared the enzyme activities measurements in assays with the only difference being the use of solvents 2% DMSO and 2% MTXE. Among the MCA substrates tested, two could not be used with MTXE: Suc-Leu-Leu-Val-Tyr-MCA (for chymotrypsin) did not dissolve in MTXE for preparation of the stock solution, and Arg-MCA (for aminopeptidase) appeared as a suspension in the 10× working solution (2 mM substrate, 20% MTXE in autoclaved artificial seawater). Figure 6 shows a comparison of hydrolytic activity for 14 MCA substrates (Table 1) in seawater samples with assay solutions of 2% DMSO and 2% MTXE and a 200 μM final substrate concentration. Hydrolytic activity was nearly the same by both methods, and the differences were not significant (*p* = 0.948 for all pairs, *n* = 42; *p* = 0.905 for aminopeptidase, *n* = 12; *p* = 0.963 for trypsin-type, *n* = 27; *p* = 0.323 for chymotrypsin-type, *n* = 3).

To assess the potential extracellular protease activities in natural seawater, the use of both 2% DMSO and 2% MTXE in assay give similar results for the hydrolysis of most MCA substrates with a final substrate concentration of 200 μM.

### Assessment of sample container material

**Figure 7:**
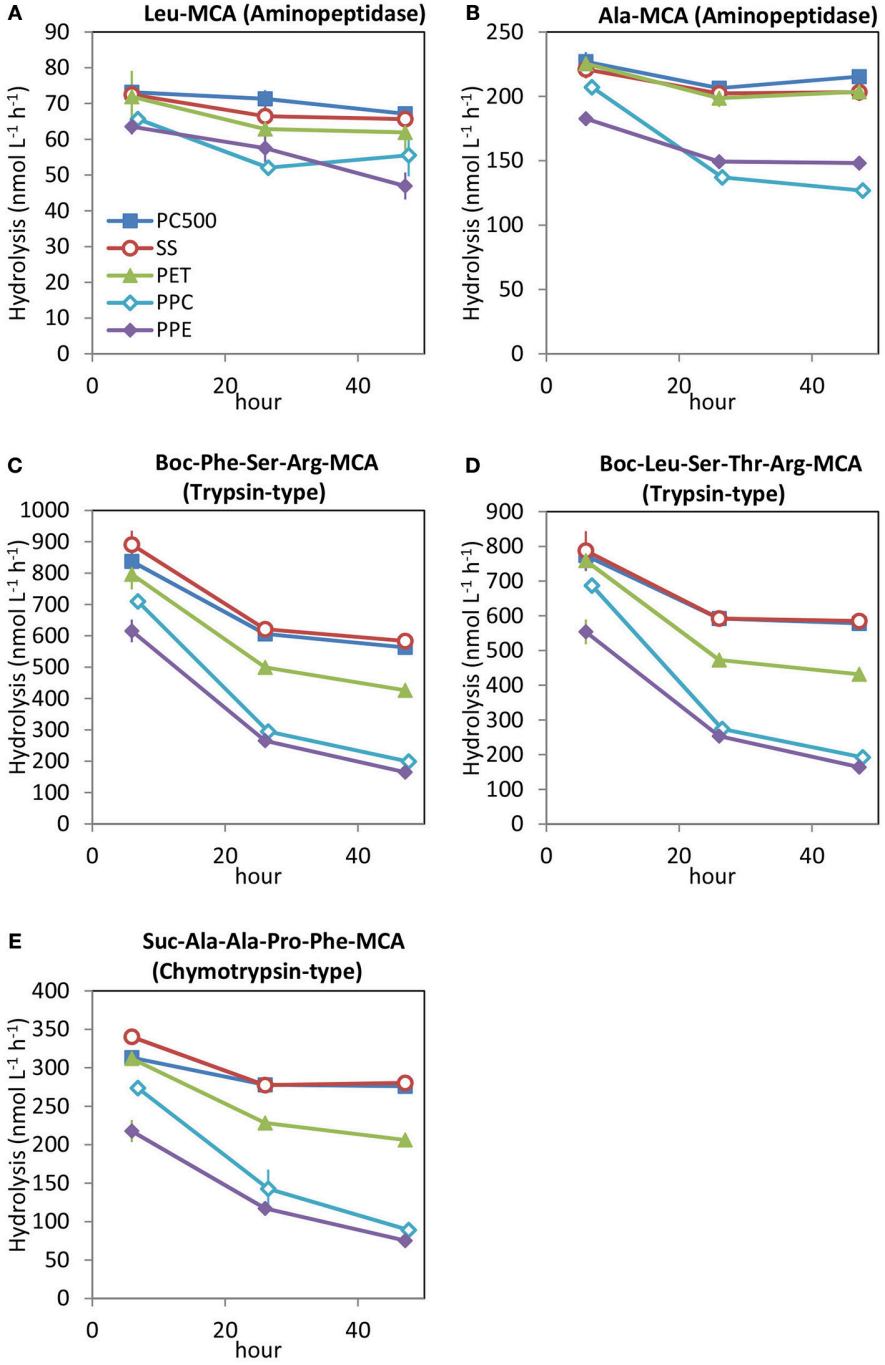
Changes in protease activities in seawater samples kept in sample containers of different materials: PC500, 500 mL polycarbonate bottle; SS, 50 mL ProteosaveSS tube; PET, 50 mL polyethylene terephthalate tube; PP, 50 mL polypropylene tube (Corning); and PPE, 50 mL polypropylene tube (Eppendorf). The time of filling the container with seawater is taken as 0 h. Hydrolysis rates of **(A)** Leu-MCA (substrate for aminopeptidase), **(B)** Ala-MCA (substrate for aminopeptidase), **(C)** Boc-Phe-Ser-Arg-MCA (substrate for trypsin), **(D)** Boc-Leu-Ser-Thr-Arg-MCA (substrate for trypsin), and **(E)** Suc-Ala-Ala-Pro-Phe-MCA (substrate for chymotrypin).

Underestimation of hydrolytic enzyme activity in seawater using regular polystyrene microplates in the assay implies that similar concern is needed for the water sample container used to store seawater samples until assay. In general, adsorption effects are less for larger volume containers. While keeping seawater samples in smaller volume containers is convenient, the sample can be easily affected by differences among materials of the containers. For samples stored five types of container materials (Figure 7), the estimated activities in seawater were different among the type of sample containers. At the first measurement, several hours after seawater collection, activity in the samples kept in 50 mL regular polypropylene tubes (PPC and PPE) was already lower than in the others, and the activities continued to decrease greatly after 1 day (at 26 h) and 2 days (at 47 h). Differences in the estimated activities of trypsin-type and chymotrypsin-type enzymes among the different tubes were greater than those of the aminopeptidase, and these trends corresponded with the effects observed for microplates (Figure 1) and from filters for size fractionation reported in Obayashi and Suzuki (2008b). The seawater sample stored in the ProteosaveSS 50 mL tube (SS), which has a hydrophilic polymer coating designed to reduce nonspecific adsorption of protein and peptide to the inside of the tube, showed similar results with the sample stored in 500 mL polycarbonate bottle (PC500), while the sample kept in the 50 mL polyethylene terephthalate tube (PET) showed lower trypsin- and chymotrypsin-type enzyme activities than water samples stored in PC500 and SS but higher than those in polypropylene tubes (PPC and PPE).

These results show that protease activity in seawater samples stored in small polypropylene tubes decreased rapidly, causing the underestimation of natural activities, especially for trypsin-type and chymotrypsin-type endopeptidases. The choice of containers for water samples is an important consideration, even when assays are conducted as soon after sampling as possible, for assessing the level of enzyme activity in natural environmental seawater samples. In general, adsorption of organic molecules onto glassware is thought to be less than that on plastics, especially if the glass surfaces are silanized. We did not test glassware in this study, however, glass vials could be thought as a good sample container, depending on the research purposes.

## Conclusions

It is thought that various enzymes in nature have a range of characteristics and behaviors in natural aquatic environments. Some enzymes in seawater are easily adsorbed onto the surfaces of some materials, and that may cause artificial effects or biases in the enzyme activities measurement. To assess the actual natural activities of microbial extracellular hydrolytic enzymes in aquatic ecosystems, materials used for both sampling and measurement assays should be carefully selected.

Water sample containers must maintain enzyme activity in the natural seawater samples for the short-term; protease activities in seawater decreased rapidly in small volume polypropylene tubes. Using fluorogenic substrates and a fluorometric microplate reader with low protein binding, black, 96-well microplates was effective for obtaining high-resolution and reliable measurements of hydrolytic enzyme activities in small volume (180 μL) of seawater samples, while regular polystyrene microplates showed significant underestimation of activities, especially for trypsin-type proteases.

For measuring the potential activities of extracellular proteases in seawater, a final substrate concentration at 200 μM in the assay (seawater sample with substrate solution) appeared to be a good saturation level for most of the tested oligopeptide analog MCA substrates for aminopeptidase, trypsin, and chymotrypsin. Stock solutions of MCA substrate are usually dissolved in a solvent, and both 1 or 2% DMSO and 2% MTXE in assay provided similar and reliable activities measurements, except for some substrates which were not sufficiently soluble. Calibration curves of AMC (product of substrate hydrolysis) should be generated under the same conditions as are used for the sample measurement. A solvent blank (sample with solvent without substrate) and an inactivated control (autoclaved seawater) should be also prepared and assayed with the samples for reliable calculation of hydrolytic enzyme activity in the sample.

Substrate concentrations at saturation level for assay of potential activity and substrate solubility may depend on sample type, targeted enzyme, and its substrate. It is important to optimize these factors for the sample types and target enzymes to obtain data that are as reliable as possible.

## Author contributions

YO designed and conducted all experiments, analyzed data, constructed discussion, and prepared the manuscript; CWB conducted portions of the experiments with YO; and SS supported all stages of the research and contributed to preparation of the manuscript.

### Conflict of interest statement

The authors declare that the research was conducted in the absence of any commercial or financial relationships that could be construed as a potential conflict of interest.
